# Machine Learning‐Based Detection of HbS and HbC Carriers in the UK General Population

**DOI:** 10.1002/jha2.70170

**Published:** 2025-11-04

**Authors:** Frederik Christensen, Deniz Kenan Kılıç, Alexander Djupnes Fuglkjær, Jesper Petersen, Tarec Christoffer El‐Galaly, Andreas Glenthøj, Jens Helby, Izabela Ewa Nielsen

**Affiliations:** ^1^ Artificial Intelligence for Operations Research Group Department of Materials and Production Aalborg University Aalborg Denmark; ^2^ R&D Department, VLMedia Ankara Türkiye; ^3^ Department of Haematology Danish Red Blood Cell Centre Copenhagen University Hospital ‐ Rigshospitalet Copenhagen Denmark; ^4^ Department of Clinical Epidemiology Aarhus University Hospital Aarhus Denmark; ^5^ Department of Molecular Medicine Aarhus University Hospital Aarhus Denmark; ^6^ Department of Hematology Aarhus University Hospital Aarhus Denmark; ^7^ Department of Clinical Medicine Aarhus University Aarhus Denmark; ^8^ Department of Clinical Medicine University of Copenhagen Copenhagen Denmark; ^9^ The Copenhagen General Population Study Copenhagen University Hospital – Herlev and Gentofte Herlev Denmark

**Keywords:** haemoglobin C disorder, haemoglobinopathies, machine learning, sickle cell disorder

## Abstract

**Background:**

Haemoglobin S (HbS) and C (HbC) are the most important sickling variants on the African continent, imposing major health burdens. Early detection of carrier status is crucial but often hindered by resource limitations.

**Objectives:**

To develop machine learning (ML) models to accurately classify HbS and HbC carriers using readily available routine blood tests, facilitating cost‐effective mass screening.

**Methods:**

We utilised demographic and routine blood parameters from 469,248 individuals from the UK general population, including 1635 individuals with HbS and/or HbC variants identified by whole exome sequencing, to develop ML models for carrier detection based on standard blood tests. Three ML models (Logistic Regression [LR], Random Forest [RF] and XGBoost [XGB]) were trained using 32 different standard blood test results.

**Results:**

All models demonstrated high discriminatory ability (ROC‐AUC: LR 0.951; RF 0.943; XGB 0.956) in the UK general population. At a sensitivity of 95%, specificities were 77% (LR), 76% (RF) and 78% (XGB). SHAP analysis revealed consistent key features across models. When use was restricted to black individuals, performance fell considerably.

**Conclusions:**

ML models based on routine blood tests effectively identify HbS and HbC carriers in a mixed general population. This approach has the potential to enhance screening efficiency by reducing reliance on specialised techniques.

## 1 Introduction

Haemoglobinopathies are among the most prevalent genetic diseases worldwide, with devastating effects on global health, especially in low‐ and middle‐income countries [1]. Hundreds of thousands of children are born every year with life‐threatening haemoglobinopathies, contributing substantially to child morbidity and mortality and imposing substantial costs. While the cost of screening and diagnosing haemoglobinopathies is manageable in many non‐endemic countries [2], there is a critical need for inexpensive and accessible diagnostic methods in resource‐limited endemic areas [3].

While thalassaemia is readily suspected in individuals with low mean cell haemoglobin (MCH) and mean cell volume (MCV), the common haemoglobin variants that may cause sickle cell disease, namely HbS and HbC, require specialised diagnostic techniques like high‐performance liquid chromatography [4]. These methods demand trained personnel, specialised medical equipment and are time‐consuming, resulting in limited access to relevant diagnostics in many areas of the world. The UK antenatal screening guidance recommends universal screening for sickle cell variants by providers with a prevalence of HbS ≥ 2% or when a structured family origin questionnaire identifies high‐risk ancestry [4]. Despite targeting screening efforts to high‐risk populations, most screened women will test negative. While haemoglobinopathy screening programs are generally considered cost‐effective, the high proportion of negative results underscores the need for more refined, data‐driven selection of individuals for diagnostic tests.

Machine learning (ML) has emerged as a powerful tool in medical diagnostics, capable of enhancing diagnostic speed, accuracy and scalability. Potentially, ML could identify complex patterns in routine blood tests indicative of haemoglobinopathies that would not be apparent to human observers. If so, such ML‐based models based on simple clinical covariates could be used for automated mass screening for HbS and HbC disorders.

In this study, we employed ML for predicting HbS and HbC carrier states in the general population individuals from the UK Biobank, including 469,248 general population individuals with genetic confirmation of carrier or non‐carrier status through whole exome sequencing. We utilise a limited set of 32 routine blood tests to determine whether HbS and HbC carriers can be effectively distinguished from non‐carriers.

## 2 Methods

### 2.1 Study Population

We studied 469,248 general population individuals aged 37–73 years from the UK Biobank, all of whom had whole exome sequencing performed as part of their enrolment in the UK Biobank [5]. The UK Biobank is a study of the general population in England, Scotland and Wales, consisting of approximately 500,000 individuals. On the day of study enrolment, participants in the UK Biobank attended a health examination where they had blood samples drawn, filled in a comprehensive questionnaire on lifestyle and health and had physical measurements obtained [6].

All participants gave written, informed consent, and the research received ethical approval from the relevant committees in the United Kingdom. The study was conducted under UK Biobank application number 99692. Study flowcharts are depicted in Figures S1 and S2.

### 2.2 Genotypes

Individuals from the UK Biobank were investigated for selected haemoglobin variants through whole exome sequencing [7]. Specifically, individuals with sickle cell trait (HbAS), sickle cell anaemia (HbSS), haemoglobin C trait (HbAC), haemoglobin C disease (HbCC) and haemoglobin SC disease (HbSC) were genetically identified. The inclusion of these specific haemoglobin (Hb) disorders enabled a focused analysis of genetic and phenotypic outcomes associated with each variant [8, 9].

Participants were separated into two groups (classes): Participants with a confirmed HbS and/or HbC gene variant, including the few individuals with two variants, were classified as individuals with HbS/C‐variants, while those without these genetic variants were designated as non‐carriers. Notably, this means that other haemoglobin variants (non‐S, non‐C) were included in the non‐carrier class.

### 2.3 Covariates Included in the Models

We obtained information on age, sex and routine blood measurements to examine whether ML models could accurately identify individuals with HbS/C‐variants based on these features. Information on age and sex was obtained from the National Health Service (NHS) Central Register [10, 11]. For blood measurements, we selected 32 routinely available blood measurements to include in the ML analyses a priori before performing our analyses. All blood parameters were measured in blood samples obtained at the date of study enrolment in the UK Biobank (included blood parameters are listed in Table 1 and Table S1).

**TABLE 1:** Baseline Characteristics of 469,248 Individuals from the UK General UK Population, presented separately for individuals with HbS/C variants and for non‐carriers. Individuals with HbS/C variants were defined as individuals with any combination of HbS and/or HbC variants (HbAS, HbSS, HbAC, HbCC or HbSC), while non‐carriers were defined as individuals without HbS or HbC variants. *p* values were calculated using logistic regression for categorical variables and linear regression for continuous variables. For ethnicity, comparisons were conducted using a one‐vs‐rest approach. no. (%) is displayed for categorical variables, while median (interquartile range) is displayed for continuous variables.no.: number. IQR: interquartile range.

	Individuals with HbS/C variants	Non‐carriers	*p* values
Individuals, no.	1635	467,613	
Men, no. (%)	716 (44)	214,232 (46)	0.10
Age, years (IQR)	51 (45–58)	58 (50–63)	2×10^−115^
Basophill count, 10^9^ cells/L (IQR)	0.03 (0.01–0.04)	0.02 (0.0–0.04)	0.002
Basophill percentage, % (IQR)	0.49 (0.33–0.74)	0.43 (0.30–0.67)	$3\times 10^{-16}$
Eosinophill count, 10^9^ cells/L (IQR)	0.12 (0.08–0.20)	0.14 (0.10–0.21)	0.003
Eosinophill percentage, % (IQR)	2.15 (1.29–3.57)	2.11 (1.37–3.27)	$2\times 10^{-8}$
Haematocrit percentage, % (IQR)	40.11 (37.34–42.74)	41.03 (38.7–43.5)	$5\times 10^{-35}$
Haemoglobin concentration, grams/decilitre (IQR)	13.58 (12.67–14.55)	14.15 (13.33–15.03)	$4\times 10^{-85}$
High light scatter reticulocyte count, 10^12^ cells/L (IQR)	0.022 (0.016–0.030)	0.016 (0.011–0.023)	$3\times 10^{-107}$
High light scatter reticulocyte percentage, % (IQR)	0.46 (0.33–0.63)	0.36 (0.25–0.504)	$5\times 10^{-44}$
Immature reticulocyte fraction, ratio (IQR)	0.35 (0.31–0.40)	0.29 (0.25–0.33)	$<1\times 10^{-308}$
Lymphocyte count, 10^9^ cells/L (IQR)	1.95 (1.55–2.42)	1.88 (1.51–2.3)	0.09
Lymphocyte percentage, % (IQR)	34.80 (29.06–41.1)	28.56 (23.90–33.50)	$2\times 10^{-225}$
Mean cell haemoglobin, picograms (IQR)	29.03 (27.61–30.43)	31.50 (30.50–32.50)	$<1\times 10^{-308}$
Mean cell haemoglobin concentration, grams/decilitre (IQR)	33.90 (33.25–34.58)	34.46 (33.89–35.10)	$1\times 10^{-104}$
Mean cell volume, femtolitres (IQR)	85.45 (81.83–88.99)	91.24 (88.58–93.9)	$<1\times 10^{-308}$
Mean platelet (thrombocyte) volume, femtolitres (IQR)	9.75 (9.03–10.63)	9.20 (8.58–9.95)	$6\times 10^{-83}$
Mean reticulocyte volume, femtolitres (IQR)	110.14 (104.98–114.59)	105.90 (101.42–110.57)	$4\times 10^{-87}$
Mean sphered cell volume, femtolitres (IQR)	83.97 (80.01–87.67)	82.68 (79.37–86.10)	$7\times 10^{-15}$
Monocyte count, 10^9^ cells/L (IQR)	0.37 (0.29–0.48)	0.45 (0.37–0.57)	$3\times 10^{-39}$
Monocyte percentage, % (IQR)	6.66 (5.20–8.11)	6.84 (5.60–8.25)	0.38
Neutrophill count, 10^9^ cells/L (IQR)	3.05 (2.35–3.90)	4.02 (3.28–4.97)	$3\times 10^{-159}$
Neutrophill percentage, % (IQR)	54.8 (47.7–61.0)	61.2 (55.7–66.5)	$3\times 10^{-194}$
Platelet count, 10^9^ cells/L (IQR)	223.6 (187.0–265.9)	248.0 (213.5–287.0)	$1\times 10^{-53}$
Platelet crit, % (IQR)	0.218 (0.187–0.253)	0.229 (0.201–0.261)	$2\times 10^{-17}$
Platelet distribution width, % (IQR)	16.34 (15.99–16.77)	16.42 (16.11–16.8)	$3\times 10^{-8}$
Red blood cell (erythrocyte) count, 10^12^ cells/L (IQR)	4.70 (4.35–5.08)	4.50 (4.23–4.79)	$3\times 10^{-83}$
Red blood cell (erythrocyte) distribution width, % (IQR)	14.25 (13.51–14.98)	13.34 (12.9–13.86)	$<1\times 10^{-308}$
Reticulocyte count, 10^12^ cells/L (IQR)	0.063 (0.048–0.080)	0.057 (0.043–0.074)	$2\times 10^{-7}$
Reticulocyte percentage, % (IQR)	1.322 (1.022–1.696)	1.258 (0.963–1.620)	0.002
White blood cell (leukocyte) count, 10^9^ cells/L (IQR)	5.69 (4.70–6.80)	6.66 (5.64–7.86)	$9\times 10^{-84}$
Alanine aminotransferase, u/L (IQR)	18.53 (14.14–25.74)	20.15 (15.42–27.42)	$9\times 10^{-8}$
Alkaline phosphatase, u/L (IQR)	79.40 (65.75–94.95)	80.40 (67.30–95.90)	0.41
Total bilirubin, µmol/L (IQR)	8.29 (6.47–11.52)	8.07 (6.42–10.42)	$4\times 10^{-13}$
Ethnicity			
Asian, no. (%)	16 (0.98)	10,522 (2.25)	$8\times 10^{-4}$
Black, no. (%)	1295 (79.20)	5957 (1.27)	$<1\times 10^{-308}$
Mixed, no. (%)	82 (5.02)	2633 (0.56)	$6\times 10^{-84}$
White, no. (%)	50 (3.06)	442,338 (94.59)	$<1\times 10^{-308}$
Unknown/missing, no. (%)	192 (11.74)	6163 (1.32)	$2\times 10^{-191}$

### 2.4 Ethnicity

To examine how our ML models performed in different ethnic groups, we obtained information from the UK Biobank's questionnaire on each individual's self‐reported ethnicity [12]. Importantly, ethnicity was only used to categorise individuals when training and testing the models in specific ethnic groups, meaning that ethnicity was not included as a variable in any of the ML models.

### 2.5 Model Development

Three supervised ML methods, Logistic Regression (LR), Random Forest (RF) and XGBoost (XGB), were employed for model training [13, 14, 15, 16]. These algorithms have shown strong potential in the field of haematology, including diagnostics of red blood cell (RBC) diseases such as haemoglobinopathies [17, 18, 19]. Furthermore, selecting methods like LR, RF and XGB provides an opportunity for greater explainability, ensuring trust in the decisions of the models, as compared to other methods (e.g., artificial neural networks).

To address high‐class imbalance, a resampling approach was applied [20, 21]. The non‐carrier group was divided into subsets, each matching the number of individuals with HbS/C variants. Each subset was then combined with the HbS/C variant group to create balanced training sets. Five‐fold cross‐validation was conducted across all training sets, with the unused non‐carrier subsets in each iteration included in the test set. This process was repeated until no noticeable change in the averaged results for each model to enhance robustness and assess model performance consistently. The full data processing, model development and model evaluation scheme can be seen in Figure S3.

### 2.6 Model Evaluation

To evaluate model performance, sensitivity, specificity and area under the receiver operating characteristic curve (ROC‐AUC) were used as primary metrics. Sensitivity, also known as recall or true positive rate, measures the model's ability to correctly identify carriers, which provides insight into how well the model detects positive cases. These metrics provide a comprehensive assessment of the model's diagnostic abilities and robustness across different classifications.

In addition, the interpretability of the model was enhanced by calculating Shapley values with the SHapley Additive exPlanations (SHAP) [22], which allowed for quantification of each feature's contribution to individual predictions. Shapley values were calculated to interpret the models and to determine the individual importance of each covariate within the model using the Shapley bar plot. A violin plot was utilised in conjunction with a colour bar to show the impact of low and high values of each covariate on the model decision.

### 2.7 Statistical Analysis

All analyses have been performed using Python (version 3.11.5) in the UK Biobank Research Analysis Platform [23].

Continuous covariates were summarised using the median and interquartile range (IQR). Categorical covariates were described as frequency counts and percentages.

We had 100% complete information on genotype, age and sex. For the 32 blood parameters that were included in our models, information on each blood parameter was missing for a mean of 3.2% (range 0.1%–5%) of individuals.

For the missing blood parameters, imputation was performed using the k‐Nearest Neighbour (k‐NN) method [24], which imputes missing values by calculating the mean of the nearest neighbours’ values, preserving data distribution and relationships among variables. To account for potential confounding factors, participants were matched on age and sex when imputing missing values. For the categorical variable ethnicity, 0.11% had missing information and 0.4% had self‐declared unknown ethnicity, and these individuals were categorised in a'Missing/unknown' category.

## 3 Results

A total of 469,248 UK Biobank participants underwent whole exome sequencing. Among them, 1635 individuals (0.3%) were genetically identified as having HbS and/or HbC variants, while 467,613 individuals (99.7%) were non‐carriers (Table 1).

Table 1 provides a summary of the baseline characteristics for individuals with HbS/C variants and non‐carriers. As expected, ethnicity varied between individuals with HbS/C variants and non‐carriers. Most individuals with HbS/C variants (79.2%) were of black ethnicity (defined as Black or Black British, Caribbean, African, or of any other black background), while non‐carriers (94.6%) predominantly belonged to the white population in the UK (defined as white British, white Irish, or any other white background).

Detailed baseline characteristics according to genotype combinations for individuals with HbS/C variants are provided in Table S2. Most individuals with HbS/C variants were heterozygous HbS (HbAS; *n* = 1258) or HbC (HbAC; *n* = 341), with only a small number of homozygous and compound heterozygous individuals, specifically HbSS (*n* = 11), HbCC (*n* = 7) and HbSC (*n* = 18).

We tested LR, XGB and RF models to identify the most suitable model for different scenarios: (1) developed and validated on the overall UK general population, (2) developed on the overall UK general population and validated specifically on the subset of Black UK general population individuals, and (3) developed and validated on the subset of Black UK general population individuals.

### 3.1 Detection of HbS and HbC Variants in the Overall UK General Population

**FIGURE 1:**
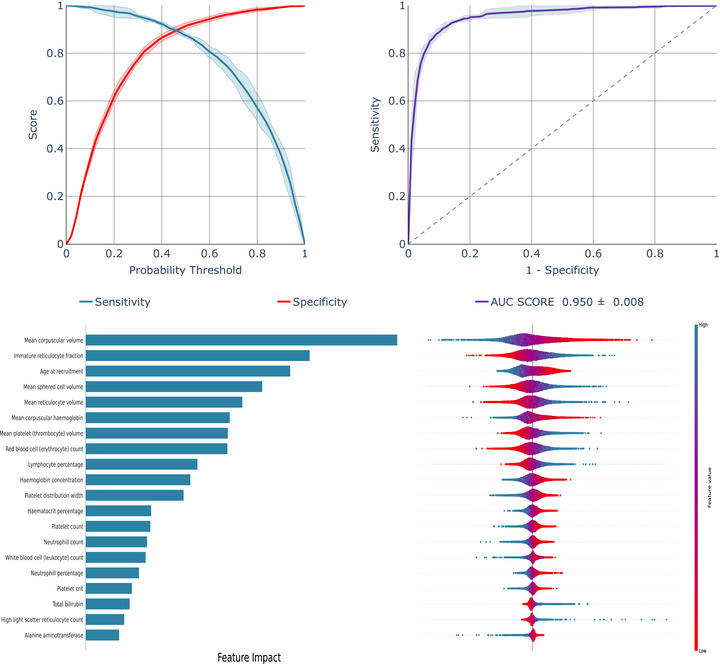
Model performance of LR model developed and validated on the overall UK general population.

In the overall UK general population, our LR model demonstrated an AUC score of 0.950 for detecting individuals with HbS/C variants (Figure 1). SHAP values highlighted that MCV, immature reticulocyte fraction, age, mean reticulocyte volume, mean sphered cell volume, RBC and mean platelet volume made important contributions to the model's predictive performance. In contrast, features like platelet crit, sex, total bilirubin and neutrophil percentages had minimal impact on the model's design. Risk and sensitivity analysis demonstrated that as sensitivity decreased from 98% to 90%, the classification thresholds rose from 16% to 44%, resulting in higher model confidence in the predictions. Meanwhile, the specificity improved from 53% to 89% (Figure 1).

The XGB model (Figure S4) had a slightly higher AUC of 0.955, while the RF model (Figure S5) had an AUC of 0.945. However, the XGB model operated at lower thresholds (2%–42%), and the RF model had lower specificities (53%–84%), making them less effective than the LR model.

The Shapley values illustrate that all three models rely on a similar set of covariates for classification, with 15 identical covariates shared between all models (see Table S3).

### 3.2 Detection of HbS and HbC Variants in Black UK General Population Individuals

To evaluate the robustness of models developed on the overall group of UK general population individuals, we constructed a subset comprising 7252 Black general population individuals from the UK Biobank (Table S4 and Figure S2). Prevalence of HbS and/or HbC variants was high in this group (1295/7, 252; 17.9%). Unlike the general UK population, no apparent differences in several covariates, including age and neutrophil count, were observed between individuals with HbS/C variants and non‐carriers within Black individuals. However, MCV remained lower in individuals with HbS/C variants (85.3 fL, IQR: 81.5–88.8) compared to non‐carriers (88.8 fL, IQR: 84.9–92.3, *p* < $1\times 10^{-308}$).

**FIGURE 2:**
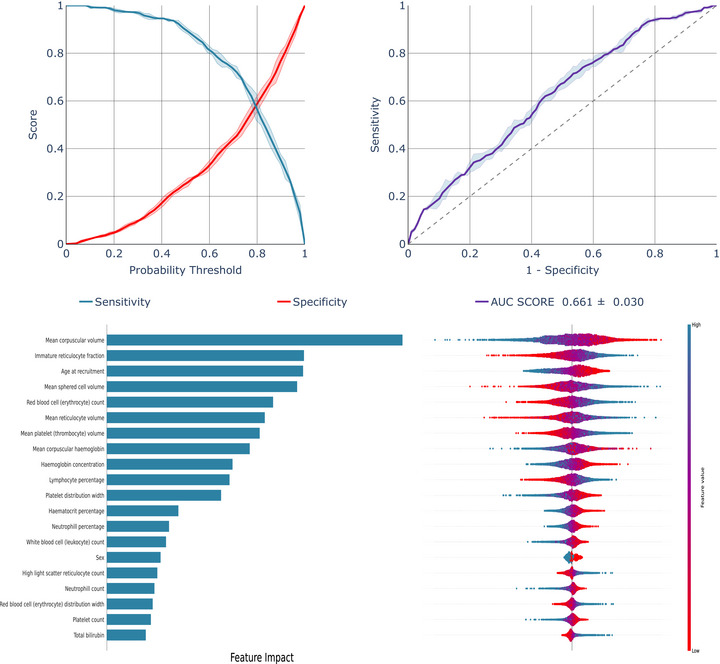
Model performance of LR model developed on the overall UK general population and validated specifically on the Black UK general population individuals.

**FIGURE 3:**
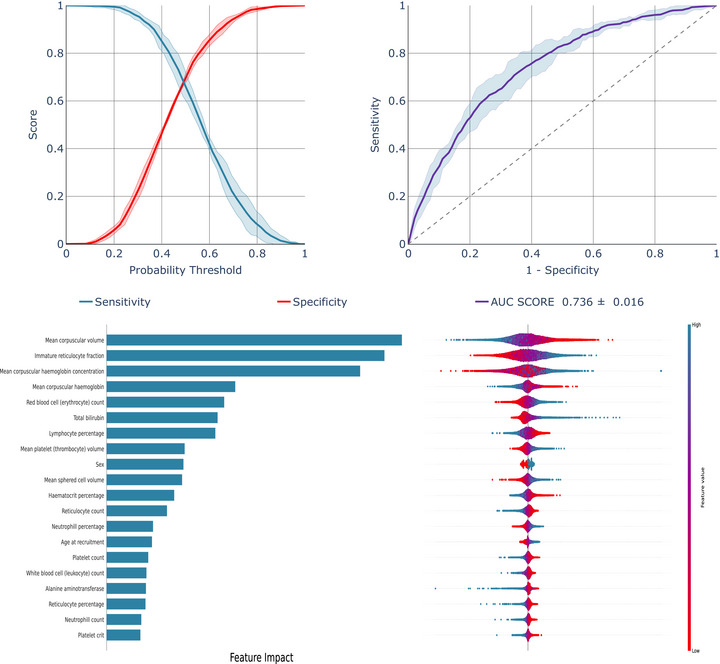
Model performance of LR model developed and validated specifically on Black UK general population individuals.

To assess the potential applicability of the algorithm in a setting where all individuals had Black ethnicity, we conducted additional analysis focusing exclusively on Black individuals: (2) ML Models developed using the entire study population and evaluated on the Black individuals specifically, and (3) models both developed and evaluated solely on the black individuals. The best performing models are shown in Figure 2 (Scenario 2) and Figure 3 (Scenario 3), with the LR model consistently outperforming others. Additional models are in Figures S6–S9.

A substantial decline in model performance was observed for Scenario 2 (Figure 2, Figures S6 and S7), only somewhat mitigated in Scenario 3, where the model was both trained and tested on black individuals (Figure 3, Figures S8 and S9). LR's ROC‐AUC improved from 0.661 in Scenario 2 to 0.736 in Scenario 3, with similar improvements for XGB and RF (Table 2). Table 2 highlights a 30%–50% drop in specificities across scenarios, highlighting reduced model effectiveness when used in the subgroup of black individuals.

**TABLE 2:** Summarised results for identifying individuals with HbS/C variants based on different fixed sensitivities (98%, 95%, 92%, 90%).

	Scenario 1: Models developed and validated on the overall UK general population											
	Logistic regression				XGBoost				Random forest			
ROC‐AUC	0.950 (± 0.008)				0.955 (± 0.006)				0.945 (± 0.009)			
Sensitivity	98%	95%	92%	90%	98%	95%	92%	90%	98%	95%	92%	90%
Threshold	16%	30%	40%	44%	2%	10%	26%	42%	20%	32%	38%	44%
Specificity	53%	78%	87%	89%	66%	78%	84%	87%	53%	73%	79%	84%
TP	1603	1554	1505	1473	1603	1554	1505	1473	1603	1554	1505	1473
FN	32	81	130	162	32	81	130	162	32	81	130	162
TN	247,836	364,739	406,824	416,177	308,626	364,739	392,796	406,824	247,836	341,358	369,415	392,796
FP	219,777	102,874	60,789	51,436	158,987	102,874	74,817	60,789	219,777	126,255	98,198	74,817

	Scenario 2: Models developed on the overall UK general population and validated specifically on Black UK general population individuals											
	Logistic regression				XGBoost				Random forest			
ROC‐AUC	0.661 (± 0.030)				0.652 (± 0.018)				0.667 (± 0.049)			
Sensitivity	98%	95%	92%	90%	98%	95%	92%	90%	98%	95%	92%	90%
Threshold	20%	36%	46%	48%	2%	14%	34%	44%	20%	28%	38%	42%
Specificity	4.9%	14.7%	23%	25%	6.5%	17.3%	24.1%	26.3%	3.3%	7.7%	15%	19.1%
TP	1270	1231	1192	1167	1270	1231	1192	1167	1270	1231	1192	1167
FN	25	64	103	128	25	64	103	128	25	64	103	128
TN	291	875	1370	1489	387	1030	1435	1566	196	458	893	1137
FP	5666	5082	4587	4468	5570	4927	4522	4391	5761	5499	5064	4820

	Scenario 3: Models developed and validated specifically on Black UK general population individuals											
	Logistic regression				XGBoost				Random forest			
ROC‐AUC	0.736 (± 0.016)				0.716 (± 0.017)				0.714 (± 0.018)			
Sensitivity	98%	95%	92%	90%	98%	95%	92%	90%	98%	95%	92%	90%
Threshold	24%	30%	34%	36%	2%	6%	8%	12%	22%	28%	32%	34%
Specificity	11%	24%	34%	39%	12%	24%	28%	34%	10%	18%	26%	31%
TP	1270	1231	1192	1167	1270	1231	1192	1167	1270	1231	1192	1167
FN	25	64	103	128	25	64	103	128	25	64	103	128
TN	656	1431	2026	2324	716	1431	1669	2026	597	1073	1550	1848
FP	5301	4526	3931	3633	5241	4526	4288	3931	5360	4884	4407	4109

SHAP analysis identified MCV and immature reticulocyte fraction as key determinants in models developed on both the overall UK general population individuals (Figure 1, Figures S4 and S5) and specifically in Black general population individuals (Figure 2, Figures S6 and S7). However, while age at recruitment and mean sphered cell volume were important predictors in the overall UK general population, MCH concentration, RBC count and bilirubin had more impact in Black individuals. Notably, excluding alanine aminotransferase, alkaline phosphatase and total bilirubin, and focusing solely on tests derived from a complete blood count, had very little impact on the models' performance (Table S5).

Finally, the ML models were developed and evaluated on the non‐Black UK general population (individuals identified as Asian or White). This resulted in a subset of 453,688 non‐Black individuals (Table S6). This resulted in an extremely low prevalence of HbS and/or HbC variants (66/453688; 0.015%).

The model performance increased slightly in performance compared to Scenario 3, with the LR's ROC‐AUC increasing to 0.768 (Table S7 and Figures S10–S12), however still not close to Scenario 1 in terms of performance.

Again, SHAP indicates that MCV and the immature reticulocyte fraction as key contributors to the model decision.

## 4 Discussion

There is an urgent need to develop cheap and reliable haemoglobinopathy screening tools. This study combined precise genetic diagnoses of haemoglobin variants with demographic data and routine blood test parameters to demonstrate the ability of ML models to classify genetically determined carriers of HbS and HbC, both of which are not readily detectable through standard blood tests.

To our knowledge, this is the largest dataset utilised for this purpose. A Dutch study included 8564 patients referred for haemoglobinopathy testing at hospital laboratories, of which 481 patients were HbS heterozygotes and 120 had sickle cell anaemia [25]. When using ML algorithms (LR and XGB) for distinguishing between adults with and without haemoglobinopathies, the Dutch study found high AUCs ≥ 0.95 for detecting α^0^‐heterozygotes, α^+^‐homozygotes and β‐thalassemia carriers, all readily identified by low MCH/MCV. However, the models showed only modest capacity to detect HbS heterozygotes, with an AUC of 0.70. The results are not directly comparable to ours, as the Dutch study included patients referred to hospital laboratories for diagnostic testing (of which some laboratories had provided information only on positive cases), whereas we studied general population individuals who were invited and genotyped based on their age and place of residence without any consideration of prior health conditions or symptoms. Furthermore, results may differ due to ethnicity, as we found ethnicity to be very important for how our models performed, while the Dutch study did not report on ethnicity. Hence, our LR model on the overall UK general population gave an AUC of 0.95, showing higher accuracy than in the Dutch study, but when applying our models specifically to black individuals only, our AUCs were comparable to those reported for detecting HbS heterozygotes in the Dutch study. Notably, while the Dutch study included information on α‐ and β‐thalassemia carrier status, their model had similar performance for detecting HbS heterozygotes as we observed when applying our model to black individuals only, suggesting that even with broader genotypic data, distinguishing HbS carriers remains challenging.

The consistent prioritisation of similar features across all models indicates underlying biological patterns associated with having HbS and/or HbC variants. Notably, features related to RBC size and haemoglobin content were key predictors, aligning with established haematological characteristics of HbS and HbC disorders [26].

The ROC‐AUC scores observed across all models demonstrate the potential utility of this approach for screening in a non‐endemic country with at‐risk minorities such as the UK.

In addition, the use of SHAP values to interpret the models improves transparency in the predictions. The SHAP values were consistent across models, showing that most of the same features were utilised for predictions. The violin plots showed a distinct separation in feature contributions between cases with low and high values, suggesting that certain feature values consistently influence model predictions.

All models prioritised the same clinically relevant features, namely MCV, MCH, mean reticulocyte volume and mean sphered cell volume, suggesting that RBC size and haemoglobin content highly impact diagnosis. In addition, a demographic feature like age appears to influence model performance across all three models.

Our study is limited by its focus on identifying only HbS and HbC, without accounting for other haemoglobinopathies, such as single‐gene deletional α‐thalassemia, which are frequently co‐inherited with HbS or HbC and can influence RBC parameters like MCV and MCH. This co‐inheritance may introduce phenotypic variability that affects model predictions. Reported frequencies of α‐thalassemia coinheritance with HbS or HbC vary widely across populations, ranging from about 17% in Mexican sickle carriers to more than half of SCD cohorts in parts of Africa [27, 28, 29, 30], underscoring that such coinheritance can be substantial and population dependent. In a study by Beutler and West differences in MCV between Black and White individuals persisted even after excluding those with iron deficiency and the common −3.7 α‐thalassaemia deletion, suggesting that other genetic factors may also influence red cell indices [27]. Importantly, this study did not investigate other α‐thalassemia deletions or β‐thalassemia, which may also be co‐inherited with HbS or HbC [28]. Further work is warranted to develop methods to detect α‐ and β‐thalassaemias in conjunction with haemoglobin variants, thereby creating a comprehensive ML algorithm.

Furthermore, ethnicity is a crucial factor, as any algorithm aimed at identifying non‐white individuals in the UK general population would likely demonstrate high sensitivity for detecting genetic conditions predominantly associated with populations of non‐White ethnic origins. When restricting our training set to black individuals only, performance was considerably reduced. This underscores the importance of considering genetic composition and its implications for diagnostic algorithms. Unfortunately, this makes the model much less suited for most countries on the African continent, where the unmet need for novel cost‐effective screening tools is much needed.

The models developed and evaluated on non‐Black individuals (Figures S8–S10) showed an increase in model performance compared to Scenario 3; however, at the cost of robustness. The small subset of individuals with HbS and/or HbC variants decreases the validity of the ML models as they do not represent the true population. However, it should be noted that the models prioritised the same set of features as for the other scenarios, showing that MCV and immature reticulocyte fraction are key features in identifying HbS and/or HbC variants.

Nonetheless, the proposed approach could be effectively implemented in populations of mixed ethnic composition, such as the UK general population, where it could serve as a triage tool to markedly reduce confirmatory testing costs. The updated 2024 UK antenatal screening guidance recommends screening all pregnant women for sickle cell variants in regions where the prevalence of HbS is ≥ 2% or when a family origin questionnaire identifies a high‐risk ancestry [4]. In this context, ML models could serve as an initial screening tool, leveraging routine blood parameters measured during pregnancy to identify individuals at risk, thereby reducing reliance on questionnaires and broad screening in designated high‐risk areas.

With each diagnostic test costing approximately £5, the implementation of ML models could potentially lower costs, as most required parameters are already part of routine blood work during pregnancy. This may make ML‐based screening particularly attractive for antenatal haemoglobinopathy programs, offering a cost‐efficient, scalable and accessible solution for early identification of at‐risk individuals.

However, a key consideration in deploying ML‐based screening is the trade‐off between sensitivity and specificity. High sensitivity is critical for ensuring that nearly all at‐risk individuals are identified, thus minimizing missed cases—a particularly important factor in antenatal care, where early detection can directly influence maternal and foetal health outcomes. Nevertheless, prioritizing sensitivity reduces specificity, leading to an increase in false positives. This, in turn, can result in unnecessary confirmatory testing, greater healthcare resource utilisation, and potentially increased anxiety for patients.

Striking the right balance depends on the specific goals of the screening program. For example, in high‐resource settings with access to widespread confirmatory testing, a higher sensitivity threshold may be favoured to ensure comprehensive detection. Conversely, in resource‐limited settings, higher specificity may be prioritised to conserve resources and reduce the burden of follow‐up testing.

## 5 Conclusion

This study suggests that ML models, when coupled with readily available routine blood tests, may offer a cost‐effective and scalable approach for screening for HbS and HbC carriers. The high accuracy and consistency across models highlight the potential of ML‐driven approaches to transform diagnostic workflows in an ethnically diverse population, but the models’ performance declined markedly when applied solely to Black individuals.

## Author Contributions

**Frederik Christensen**: concept and design, collection of data, data analysis, interpretation of results, manuscript first draft, manuscript revision. **Deniz Kenan Kılıç**: concept and design, interpretation of results, manuscript revision. **Alexander Djupnes Fuglkjær**: interpretation of results, manuscript revision. **Jesper Petersen**: interpretation of results, manuscript revision. **Tarec Christoffer El‐Galaly**: concept and design, interpretation of results, manuscript revision. **Andreas Glenthøj**: concept and design, data analysis, interpretation of results, manuscript revision. **Jens Helby**: concept and design, collection of data, data analysis, interpretation of results, manuscript revision. **Izabela Ewa Nielsen**: concept and design, interpretation of results, manuscript revision. All authors approve of the final manuscript.

## Funding

This research was supported by the Novo Nordisk Foundation [grant number NNF22OC0078228].

## Conflicts of Interest

**Andreas Glenthøj**: Agios, Novo Nordisk, Pharmacosmos, Vertex Pharmaceuticals (Consultancy / Advisory board) and Agios, Bristol Myers Squibb, Novo Nordisk, Saniona, Sanofi (Research funding/support). **Jens Helby**: Sanofi (research funding, conference travel grant, advisory board), Disc Medicine (advisory board). The other authors declare no conflicts of interest.

## Supporting information




**Supporting File 1**: jha270170‐sup‐0001‐figureS1.png

**Supporting File 2**: jha270170‐sup‐0002‐figureS2.png

**Supporting File 3**: jha270170‐sup‐0003‐figureS3.png

**Supporting File 4**: jha270170‐sup‐0004‐SuppMat.docx

## Data Availability

This research has been conducted using data from UK Biobank (Application Number 99692), a publicly accessible biomedical database. The data are available upon application to UK Biobank, subject to approval by the UK Biobank Access Management Team. The authors are not permitted to share the data directly due to the terms of the UK Biobank Material Transfer Agreement. Researchers wishing to access the data can apply via the UK Biobank Access Management System. Complete code used in this analysis, including data pre‐processing and model training scripts, can be provided upon reasonable request from the corresponding author.
