# Supplementary figures and images for: Machine Learning‐Based Detection of HbS and HbC Carriers in the UK General Population
Source: EJHaem. 2025 Nov 4;6(6):e70170. doi: 10.1002/jha2.70170 (PMC12584039; doi:10.1002/jha2.70170)

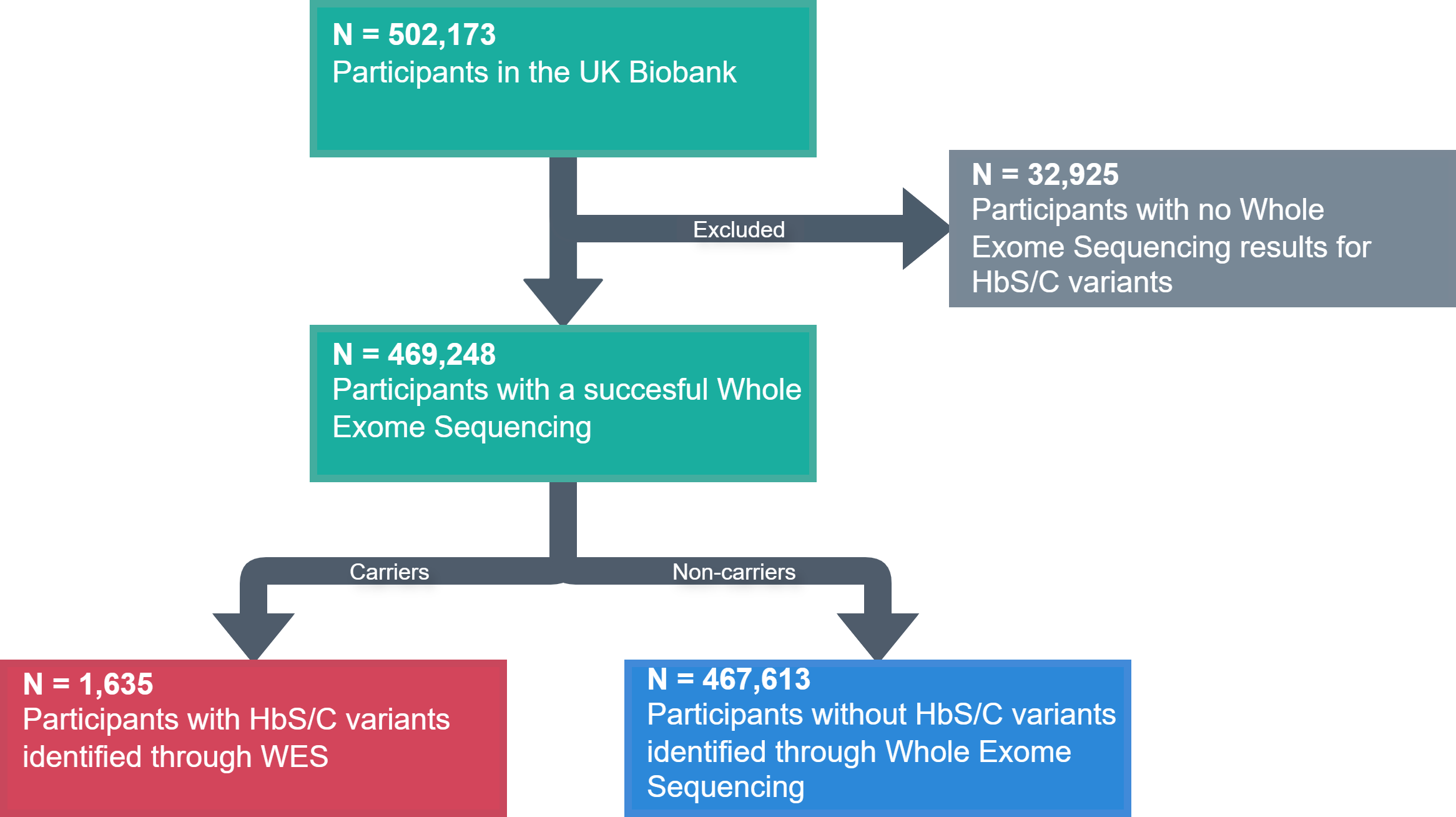

Supplement: Supplementary file 1 — Supporting File 1: jha270170‐sup‐0001‐figureS1.png [file JHA2-6-e70170-s001.png]

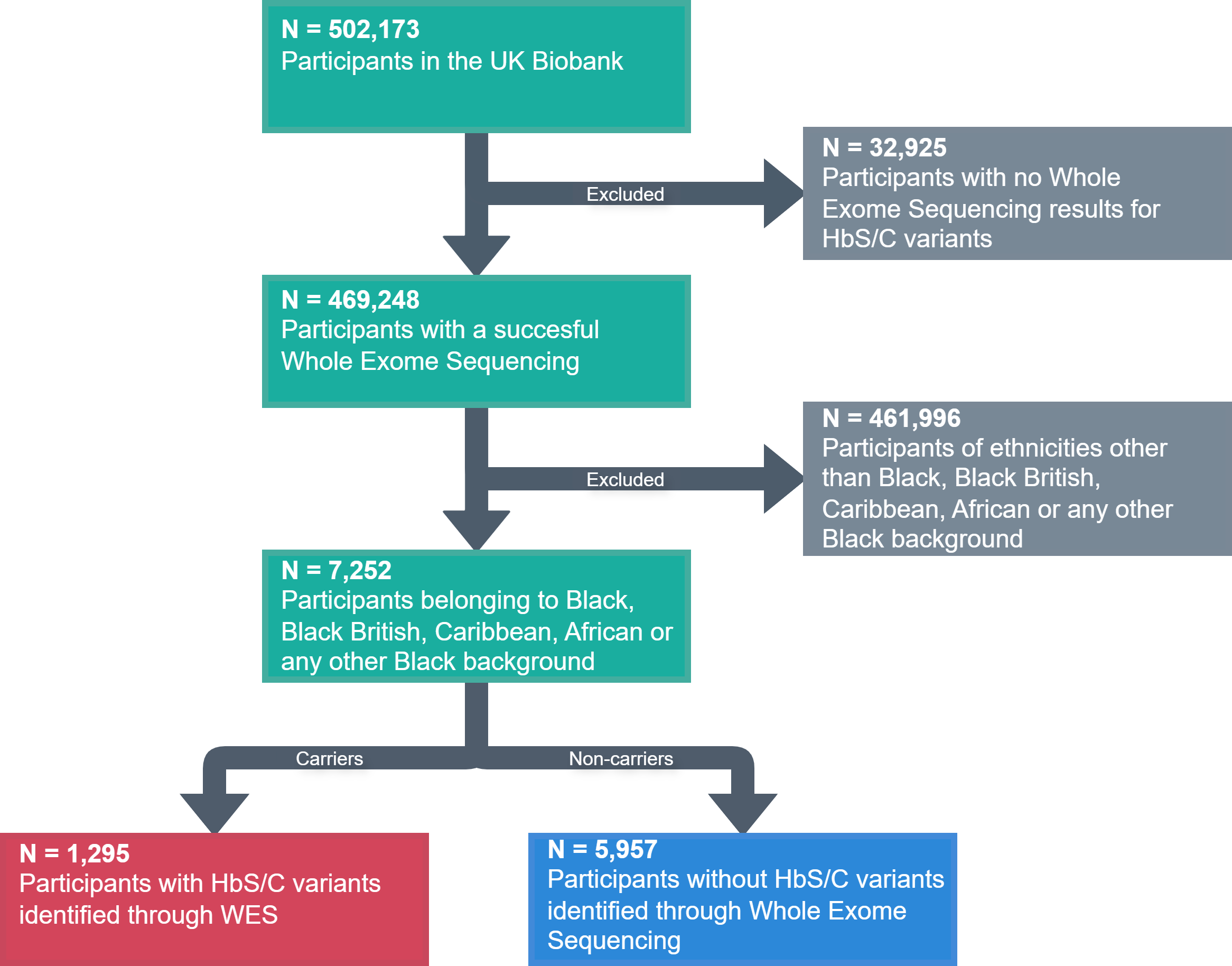

Supplement: Supplementary file 2 — Supporting File 2: jha270170‐sup‐0002‐figureS2.png [file JHA2-6-e70170-s003.png]

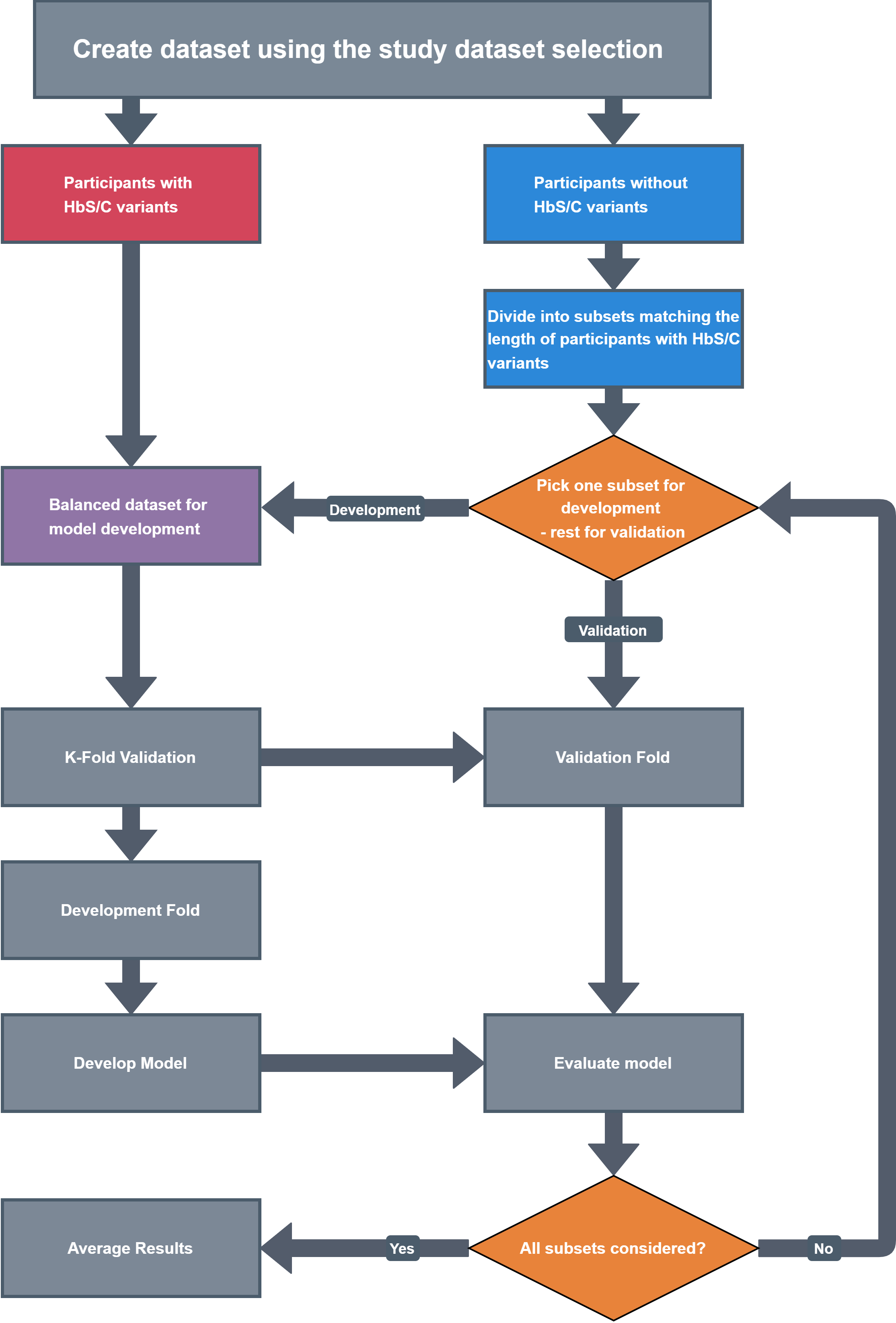

Supplement: Supplementary file 3 — Supporting File 3: jha270170‐sup‐0003‐figureS3.png [file JHA2-6-e70170-s004.png]
