# Supplementary material for: Machine Learning‐Based Detection of HbS and HbC Carriers in the UK General Population
Source: EJHaem. 2025 Nov 4;6(6):e70170. doi: 10.1002/jha2.70170 (PMC12584039; doi:10.1002/jha2.70170)
Supplement: Supplementary file 4 — Supporting File 4: jha270170‐sup‐0004‐SuppMat.docx [file JHA2-6-e70170-s002.docx]

# Title: Machine Learning-Based Detection of HbS and HbC Carriers in the UK General Population

# Supplementary Material

## Covariates

| Demographic Information (obtained at study enrolment) | Sex  Age |
| --- | --- |
| Blood samples (obtained at study enrolment) | Basophill count  Basophill percentage  Eosinophill count  Eosinophill percentage  Haematocrit percentage  Haemoglobin concentration  High light scatter reticulocyte count  High light scatter reticulocyte percentage  Immature reticulocyte fraction  Lymphocyte count  Lymphocyte percentage  Mean cell haemoglobin  Mean cell haemoglobin concentration  Mean cell volume  Mean platelet (thrombocyte) volume  Mean reticulocyte volume  Mean sphered cell volume  Monocyte count  Monocyte percentage  Neutrophill count  Neutrophill percentage  Platelet count  Platelet crit  Platelet distribution width  Red blood cell (erythrocyte) count  Red blood cell (erythrocyte) distribution width  Reticulocyte count  Reticulocyte percentage  White blood cell (leukocyte) count  Alanine aminotransferase  Alkaline phosphatase  Total bilirubin |
| Genotype | Haemoglobin C trait (Hb AC)  Haemoglobin C disease (Hb C)  Sickle cell trait (Hb AS)  Sickle cell disease (Hb S)  Haemoglobin SC disease (Hb SC) |

***Supplementary Table 1:*** *Covariate features considered in the ML models.*

## Baseline characteristics according to genotype combinations for individuals with HbS or HbC variants

|  | HbAS | HbSS | HbAC | HbCC | HbSC |
| --- | --- | --- | --- | --- | --- |
| Individuals, no. | 1,258 | 11 | 341 | 7 | 18 |
| Men, no. (%) | 547 (44) | 7 (64) | 149 (44) | <5 | 10 (56) |
| Age, years (IQR) | 51 (45-58) | 51 (46-54) | 51 (45-59) | 51 (47-58) | 49 (44-57) |
| Basophill count, 10^9 cells/litre (IQR) | 0.02 (0.01-0.04) | 0.03 (0.02-0.05) | 0.03 (0.02-0.05) | 0.04 (0.01-0.05) | 0.03 (0.01-0.05) |
| Basophill percentage, % (IQR) | 0.48 (0.33-0.73) | 0.46 (0.28-0.61) | 0.50 (0.35-0.8) | 0.60 (0.45-0.62) | 0.50 (0.38-0.70) |
| Eosinophill count, 10^9 cells/litre (IQR) | 0.12 (0.08-0.20) | 0.13 (0.10-0.20) | 0.12 (0.07-0.20) | 0.29 (0.24-0.30) | 0.18 (0.12-0.23) |
| Eosinophill percentage, % (IQR) | 2.17 (1.3-3.63) | 2.15 (1.78-2.39) | 2.06 (1.20-3.27) | 3.61 (3.28-4.28) | 2.52 (2.01-2.85) |
| Haematocrit percentage, % (IQR) | 40.10 (37.40-42.65) | 29.56 (24.76-37.04) | 40.46 (37.48-43.27) | 34.95 (32.55-40.06) | 33.98 (31.11-40.05) |
| Haemoglobin concentration, grams/decilitre (IQR) | 13.52 (12.66-14.47) | 9.93 (8.27-12.66) | 13.82 (12.9-14.87) | 11.85 (11.64-13.49) | 11.59 (10.96-13.89) |
| High light scatter reticulocyte count, 10^12 cells/litre (IQR) | 0.021 (0.015-0.029) | 0.053 (0.034-0.088) | 0.024 (0.016-0.032) | 0.077 (0.056-0.109) | 0.057 (0.049-0.088) |
| High light scatter reticulocyte percentage, % (IQR) | 0.45 (0.32-0.62) | 1.61 (0.73-2.88) | 0.49 (0.34-0.64) | 1.42 (1.15-2.18) | 1.55 (1.05-1.82) |
| Immature reticulocyte fraction, ratio (IQR) | 0.34 (0.30-0.39) | 0.50 (0.47-0.56) | 0.37 (0.31-0.42) | 0.41 (0.39-0.47) | 0.49 (0.44-0.50) |
| Lymphocyte count, 10^9 cells/litre (IQR) | 1.88 (1.51-2.3) | 1.92 (1.80-2.51) | 2.23 (1.8-2.80) | 2.64 (2.25-2.78) | 1.99 (1.67-2.30) |
| Lymphocyte percentage, % (IQR) | 34.20 (28.61-40.12) | 34.46 (24.87-41.89) | 37.62 (30.79-44.22) | 34.68 (31.79-37.63) | 33.46 (29.13-37.77) |
| Mean cell haemoglobin, picograms (IQR) | 29.05 (27.7-30.50) | 30.99 (27.39-31.51) | 29.00 (27.36-30.05) | 25.11 (24.89-25.72) | 29.12 (27.80-31.86) |
| Mean cell haemoglobin concentration, grams/decilitre (IQR) | 33.80 (33.16-34.42) | 34.17 (33.40-34.50) | 34.32 (33.69-35.00) | 33.89 (33.72-34.03) | 34.81 (34.34-34.99) |
| Mean cell volume, femtolitres (IQR) | 85.90 (82.40-89.50) | 89.47 (80.19-93.71) | 83.89 (80.33-87.18) | 74.56 (73.53-75.77) | 82.31 (79.83-89.41) |
| Mean platelet (thrombocyte) volume, femtolitres (IQR) | 9.75 (9.00-10.63) | 9.70 (9.46-9.85) | 9.72 (9.08-10.56) | 10.48 (10.05-10.85) | 9.82 (9.54-10.82) |
| Mean reticulocyte volume, femtolitres (IQR) | 110.55 (105.50-114.81) | 124.36 (118.92-129.04) | 108.66 (104.00-113.44) | 101.56 (99.70-105.21) | 113.93 (110.62-117.51) |
| Mean sphered cell volume, femtolitres (IQR) | 84.09 (80.21-87.80) | 91.46 (84.97-96.59) | 83.33 (79.53-87.23) | 71.65 (65.74-78.27) | 84.29 (80.77-88.03) |
| Monocyte count, 10^9 cells/litre (IQR) | 0.37 (0.29-0.47) | 0.56 (0.45-0.74) | 0.40 (0.30-0.52) | 0.39 (0.29-0.53) | 0.27 (0.21-0.43) |
| Monocyte percentage, % (IQR) | 6.65 (5.21-8.11) | 8.66 (7.76-10.81) | 6.74 (5.18-8.10) | 5.82 (4.05-6.97) | 4.36 (3.69-7.25) |
| Neutrophill count, 10^9 cells/litre (IQR) | 3.00 (2.32-3.88) | 3.84 (2.78-4.45) | 3.09 (2.39-3.88) | 4.10 (3.89-4.30) | 3.93 (3.21-4.43) |
| Neutrophill percentage, % (IQR) | 55.30 (48.90-61.50) | 52.55 (46.13-68.00) | 51.90 (45.10-58.90) | 54.50 (52.60-57.00) | 57.90 (49.00 -63.93) |
| Platelet count, 10^9 cells/litre (IQR) | 221.5 (186.0-263.6) | 235.8 (181.9-358.5) | 230.4 (195.9-273.0) | 204.4 (123.3-283.4) | 151.9 (130.8-239.7) |
| Platelet crit, % (IQR) | 0.215 (0.186-0.252) | 0.235 (0.171-0.346) | 0.227 (0.196-0.256) | 0.204 (0.127-0.276) | 0.1615 (0.138-0.220) |
| Platelet distribution width, % (IQR) | 16.34 (15.99-16.76) | 16.08 (15.74-16.80) | 16.31 (15.96-16.77) | 16.48 (16.34-16.52) | 17.29 (16.51-17.81) |
| Red blood cell (erythrocyte) count, 10^12 cells/litre (IQR) | 4.66 (4.33-5.03) | 3.41 (2.63-4.63) | 4.86 (4.49-5.24) | 4.63 (4.21-5.38) | 4.17 (3.81-4.60) |
| Red blood cell (erythrocyte) distribution width, % (IQR) | 14.12 (13.44-14.84) | 18.83 (16.06-20.02) | 14.53 (13.9-15.2) | 19.43 (17.54-21.33) | 17.11 (16.19-19.35) |
| Reticulocyte count, 10^12 cells/litre (IQR) | 0.061 (0.047-0.079) | 0.114 (0.069-0.178) | 0.065 (0.050-0.081) | 0.177 (0.141-0.230) | 0.138 (0.109-0.167) |
| Reticulocyte percentage, % (IQR) | 1.313 (1.010-1.691) | 3.412 (1.532-5.620) | 1.328 (1.056-1.657) | 3.351 (2.757-5.091) | 3.272 (2.596-3.527) |
| White blood cell (leukocyte) count, 10^9 cells/litre (IQR) | 5.52 (4.66-6.60) | 6.29 (5.73-8.35) | 6.00 (5.00-7.28) | 7.15 (6.95-8.02) | 6.08 (5.86-6.99) |
| Alanine aminotransferase, u/l (IQR) | 18.27 (13.95-25.56) | 14.65 (11.12-19.68) | 19.73 (14.82-26.23) | 17.35 (13.75-23.95) | 19.74 (14.67-23.19) |
| Alkaline phosphatase, u/l (IQR) | 79.40 (65.5-95.1) | 106.10 (80.20-129.55) | 79.15 (66.7-93.78) | 71.85 (65.30-85.15) | 86.35 (74.43-98.75) |
| Total bilirubin, umol/l (IQR) | 8.11 (6.40-11.03) | 35.88 (28.19-43.39) | 8.62 (6.52-11.88) | 23.20 (13.99-48.90) | 17.10 (13.27-21.19) |

***Supplementary Table 2:*** *Baseline characteristics of 1,635 individuals from the general population according to genotype combinations for individuals with HbS or HbC variants. Due to data privacy regulations, "<5" is reported for table cells with fewer than five individuals; Similarly, the number of individuals with each genotype in specific ethnic groups were too low to be published in accordance with data privacy regulations, and ethnicity is therefore not included in this table.no. (%) is displayed for categorical variables while median (interquartile range) is displayed for continuous variables.
no.: number. IQR: interquartile range.*

## Covariate Importance Across Models

| Covariate | Model ranking | | |
| --- | --- | --- | --- |
|  | **Logistic Regression** | **XGBoost** | **Random Forest** |
| Age at recruitment | 3 | 6 | 7 |
| Haemoglobin concentration | 10 | 20 | 16 |
| Immature reticulocyte fraction | 2 | 5 | 3 |
| Lymphocyte percentage | 9 | 12 | 9 |
| Mean corpuscular haemoglobin | 6 | 1 | 1 |
| Mean corpuscular volume | 1 | 2 | 2 |
| Mean Platelet volume | 7 | 9 | 17 |
| Mean reticulocyte volume | 5 | 4 | 4 |
| Mean sphered cell volume | 4 | 3 | 6 |
| Neutrophil count | 14 | 7 | 5 |
| Neutrophil percentage | 16 | 15 | 10 |
| Platelet Count | 13 | 10 | 15 |
| Platelet Distribution width | 11 | 11 | 20 |
| Red blood cell count | 8 | 18 | 18 |
| White blood cell count | 15 | 19 | 12 |

***Supplementary Table 3:*** *Shared important covariates (15) shared across all three ML models, as identified by Shapley value analysis, indicating consistent reliance on these features for classification.*

## Baseline characteristics of the black UK general population individuals

|  | Individuals with HbS/C variants | Non-Carriers | P-values |
| --- | --- | --- | --- |
| Individuals, no. | 1,295 | 5,957 |  |
| Men, no. (%) | 568 (44) | 2,550 (43) | 0.49 |
| Age, years (IQR) | 51 (45 -58) | 50 (45-58) | 0.89 |
| Basophill count, 10^9 cells/litre (IQR) | 0.03 (0.01-0.04) | 0.02 (0.01-0.04) | 0.009 |
| Basophill percentage, % (IQR) | 0.49 (0.33-0.75) | 0.47 (0.32-0.70) | 0.007 |
| Eosinophill count, 10^9 cells/litre (IQR) | 0.12 (0.08-0.20) | 0.11 (0.07-0.20) | 0.34 |
| Eosinophill percentage, % (IQR) | 2.17 (1.30-3.60) | 2.09 (1.27-3.45) | 0.10 |
| Haematocrit percentage, % (IQR) | 40.05 (37.27-42.73) | 40.06 (37.43-43.00) | 0.03 |
| Haemoglobin concentration, grams/decilitre (IQR) | 13.53 (12.60-14.52) | 13.51 (12.60-14.56) | 0.54 |
| High light scatter reticulocyte count, 10^12 cells/litre (IQR) | 0.022 (0.015-0.030) | 0.020 (0.014-0.027) | $3\times{10}^{-13}$ |
| High light scatter reticulocyte percentage, % (IQR) | 0.46 (0.33-0.63) | 0.44 (0.31-0.59) | $2\times{10}^{-8}$ |
| Immature reticulocyte fraction, ratio (IQR) | 0.35 (0.31-0.40) | 0.32 (0.28-0.37) | $3\times{10}^{-38}$ |
| Lymphocyte count, 10^9 cells/litre (IQR) | 1.95 (1.57-2.40) | 2.05 (1.67-2.50) | $2\times{10}^{-8}$ |
| Lymphocyte percentage, % (IQR) | 35.43 (29.73-41.69) | 37.37 (31.16-43.60) | $7\times{10}^{-10}$ |
| Mean cell haemoglobin, picograms (IQR) | 28.92 (27.56-30.40) | 30.01 (28.40-31.45) | $1\times{10}^{-33}$ |
| Mean cell haemoglobin concentration, grams/decilitre (IQR) | 33.85 (33.21-34.54) | 33.70 (33.06-34.32) | $2\times{10}^{-6}$ |
| Mean cell volume, femtolitres (IQR) | 85.30 (81.54-88.84) | 88.76 (84.90-92.34) | $2\times{10}^{-61}$ |
| Mean platelet (thrombocyte) volume, femtolitres (IQR) | 9.78 (9.02-10.63) | 9.54 (8.84-10.37) | $8\times{10}^{-7}$ |
| Mean reticulocyte volume, femtolitres (IQR) | 110.34 (105.39-114.80) | 110.74 (105.75-115.95) | 0.03 |
| Mean sphered cell volume, femtolitres (IQR) | 84.07 (80.09-87.80) | 85.00 (81.11-89.18) | $1\times{10}^{-8}$ |
| Monocyte count, 10^9 cells/litre (IQR) | 0.37 (0.29-0.48) | 0.38 (0.30-0.47) | 0.76 |
| Monocyte percentage, % (IQR) | 6.73 (5.23-8.17) | 6.62 (5.30-8.13) | 0.36 |
| Neutrophill count, 10^9 cells/litre (IQR) | 2.94 (2.30-3.76) | 2.84 (2.20-3.72) | 0.42 |
| Neutrophill percentage, % (IQR) | 53.95 (46.93-60.30) | 52.05 (45.51-58.90) | $3\times{10}^{-6}$ |
| Platelet count, 10^9 cells/litre (IQR) | 220.30 (185.60-264.05) | 231.00 (196.10-271.95) | $5\times{10}^{-6}$ |
| Platelet crit, % (IQR) | 0.216 (0.185-0.252) | 0.221 (0.192-0.253) | 0.005 |
| Platelet distribution width, % (IQR) | 16.32 (15.99-16.77) | 16.28 (15.92-16.67) | $5\times{10}^{-5}$ |
| Red blood cell (erythrocyte) count, 10^12 cells/litre (IQR) | 4.70 (4.35-5.08) | 4.54 (4.21-4.90) | $2\times{10}^{-19}$ |
| Red blood cell (erythrocyte) distribution width, % (IQR) | 14.27 (13.54-15.02) | 13.90 (13.28-14.63) | $3\times{10}^{-17}$ |
| Reticulocyte count, 10^12 cells/litre (IQR) | 0.063 (0.048-0.080) | 0.061 (0.047-0.078) | 0.04 |
| Reticulocyte percentage, % (IQR) | 1.316 (1.020-1.696) | 1.339 (1.035-1.720) | 0.93 |
| White blood cell (leukocyte) count, 10^9 cells/litre (IQR) | 5.59 (4.65-6.59) | 5.59 (4.66-6.73) | 0.12 |
| Alanine aminotransferase, u/l (IQR) | 18.57 (14.14-25.89) | 19.28 (14.78-26.41) | 0.07 |
| Alkaline phosphatase, u/l (IQR) | 79.40 (65.45-95.15) | 80.60 (66.75-96.85) | 0.04 |
| Total bilirubin, umol/l (IQR) | 8.26 (6.46-11.62) | 7.62 (5.85-10.29) | $2\times{10}^{-16}$ |

***Supplementary Table 3:*** *Baseline characteristics of 7,252 black UK general population individuals, presented separately for individuals with HbS/C variants and for non-carriers. Individuals with HbS/C variants were defined as individuals with any combination of HbS and/or HbC variants (HbAS, HbSS, HbAC, HbCC, or HbSC), while non-carriers were defined as individuals without HbS or HbC variants. P-values were calculated using logistic regression for categorical variables and linear regression for continuous variables. no. (%) is displayed for categorical variables while median (interquartile range) is displayed for continuous variables.
no.: number. IQR: interquartile range.*

## Model results for each scenario without including alanine aminotransferase, alkaline phosphatase and total bilirubin in the models.

|  | Scenario 1: Models developed and validated on the overall UK general population | | | | | | | | | | | | | | | | | | |
| --- | --- | --- | --- | --- | --- | --- | --- | --- | --- | --- | --- | --- | --- | --- | --- | --- | --- | --- | --- |
|  | **Logistic Regression** | | | | **XGBoost** | | | | | | | **Random Forest** | | | | | | | |
| ROC-AUC | 0.951 (±0.004) | | | | 0.951 (±0.004) | | | | | | | 0.943 (±0.005) | | | | | | | |
| Sensitivity | 98 % | 95 % | 92 % | 90 % | 98 % | 95 % | | 92 % | | 90 % | | 98 % | | 95 % | | 92 % | | 90 % | |
| Threshold | 10 % | 26 % | 36 % | 44 % | 2 % | 8 % | | 26 % | | 40 % | | 18 % | | 32 % | | 40 % | | 44 % | |
| Specificity | 58 % | 79 % | 85 % | 88 % | 65 % | 77 % | | 85 % | | 87 % | | 53 % | | 74 % | | 82 % | | 85 % | |
| TP | 1603 | 1554 | 1505 | 1473 | 1603 | 1554 | | 1505 | | 1473 | | 1603 | | 1554 | | 1505 | | 1473 | |
| FN | 32 | 81 | 130 | 162 | 32 | 81 | | 130 | | 162 | | 32 | | 81 | | 130 | | 162 | |
| TN | 273076 | 368384 | 395942 | 411345 | 305541 | 359104 | | 395826 | | 408734 | | 248976 | | 347755 | | 383355 | | 397133 | |
| FP | 194537 | 99229 | 71671 | 56268 | 162072 | 108509 | | 71787 | | 58879 | | 218637 | | 119858 | | 84258 | | 70480 | |
|  | **Scenario 2: Models developed on the overall UK general population and validated specifically on Black UK general population individuals** | | | | | | | | | | | | | | | | | | |
|  | **Logistic Regression** | | | | **XGBoost** | | | | | | | **Random Forest** | | | | | | | |
| ROC-AUC | 0.646 (±0.016) | | | | 0.653 (±0.017) | | | | | | | 0.645 (±0.018) | | | | | | | |
| Sensitivity | 98 % | 95 % | 92 % | 90 % | 98 % | 95 % | | 92 % | | 90 % | | 98 % | | 95 % | | 92 % | | 90 % | |
| Threshold | 20 % | 34 % | 46 % | 54 % | 2 % | 16 % | | 42 % | | 56 % | | 24 % | | 38 % | | 44 % | | 50 % | |
| Specificity | 10 % | 16 % | 22 % | 26 % | 7 % | 16 % | | 24 % | | 27 % | | 7 % | | 16 % | | 20 % | | 26 % | |
| TP | 1270 | 1231 | 1192 | 1167 | 1270 | 1231 | | 1192 | | 1167 | | 1270 | | 1231 | | 1192 | | 1167 | |
| FN | 25 | 64 | 103 | 128 | 25 | 64 | | 103 | | 128 | | 25 | | 64 | | 103 | | 128 | |
| TN | 569 | 964 | 1306 | 1546 | 431 | 970 | | 1401 | | 1607 | | 408 | | 927 | | 1216 | | 1547 | |
| FP | 5388 | 4993 | 4651 | 4411 | 5526 | 4987 | | 4556 | | 4350 | | 5549 | | 5030 | | 4741 | | 4410 | |
|  | **Scenario 3: Models developed and validated specifically on Black UK general population individuals** | | | | | | | | | | | | | | | | | | |
|  | **Logistic Regression** | | | | **XGBoost** | | | | | | | | **Random Forest** | | | | | | |
| ROC-AUC | 0.731 (±0.011) | | | | 0.697 (±0.010) | | | | | | | | 0.701 (±0.011) | | | | | | |
| Sensitivity | 98 % | 95 % | 92 % | 90 % | 98 % | | 95 % | | 92 % | | 90 % | | 98 % | | 95 % | | 92 % | | 90 % |
| Threshold | 20 % | 26 % | 30 % | 32 % | 2 % | | 4 % | | 8 % | | 10 % | | 20 % | | 26 % | | 32 % | | 34 % |
| Specificity | 11 % | 22 % | 30 % | 34 % | 10 % | | 17 % | | 26 % | | 30 % | | 8 % | | 17 % | | 28 % | | 32 % |
| TP | 1270 | 1231 | 1192 | 1167 | 1270 | | 1231 | | 1192 | | 1167 | | 1270 | | 1231 | | 1192 | | 1167 |
| FN | 25 | 64 | 103 | 128 | 25 | | 64 | | 103 | | 128 | | 25 | | 64 | | 103 | | 128 |
| TN | 667 | 1291 | 1770 | 2006 | 622 | | 1031 | | 1577 | | 1779 | | 496 | | 991 | | 1651 | | 1894 |
| FP | 5290 | 4666 | 4187 | 3951 | 5335 | | 4926 | | 4380 | | 4178 | | 5461 | | 4966 | | 4306 | | 4063 |

***Supplementary Table 5: Summarized Results for identifying individuals with HbS/C variants Based on Different Fixed Sensitivities (98, 95, 92, 90 %) without including alanine aminotransferase, alkaline phosphatase and total bilirubin in the models.*** *This table summarizes the performance of Logistic Regression, XGBoost, and Random Forest models at specified sensitivities for different scenarios. Metrics include the corresponding probability threshold, specificity, and the number of true positives (TP), false negatives (FN), true negatives (TN), and false positives (FP) for each model. The table highlights variations in specificity and risk threshold across models and sensitivity levels. The probability threshold is the probability value that serves as the cut-off for a model's predictions. It determines when the model assigns a specific class label based on its probability estimates. If the probability falls below the threshold, the individual is classified as a non-carrier. Conversely, if it exceeds the threshold, the model predicts that the individual carries HbS/C variants.*

1. Baseline characteristics of the non-black UK general population individuals

|  | Individuals with HbS/C variants | Non-Carriers | P-values |
| --- | --- | --- | --- |
| Individuals, no. | 66 | 453622 |  |
| Men, no. (%) | 38 (58) | 208108 (46) | 0.06 |
| Age, years (IQR) | 56 (48-62) | 58.00 (50.00-63.00) | 0.06 |
| Basophill count, 10^9 cells/litre (IQR) | 0.02 (0.00-0.04) | 0.02 (0.00-0.04) | 0.58 |
| Basophill percentage, % (IQR) | 0.40 (0.30-0.60) | 0.43 (0.30-0.67) | 0.47 |
| Eosinophill count, 10^9 cells/litre (IQR) | 0.14 (0.10-0.21) | 0.14 (0.10-0.21) | 0.37 |
| Eosinophill percentage, % (IQR) | 1.81 (1.20-3.31) | 2.11 (1.37-3.27) | 0.48 |
| Haematocrit percentage, % (IQR) | 41.52 (38.93-43.39) | 41.05 (38.70-43.50) | 0.71 |
| Haemoglobin concentration, grams/decilitre (IQR) | 14.47 (13.48-15.02) | 14.17 (13.35-15.04) | 0.73 |
| High light scatter reticulocyte count, 10^12 cells/litre (IQR) | 0.02 (0.02-0.03) | 0.02 (0.01-0.02) | $2\times{10}^{-4}$ |
| High light scatter reticulocyte percentage, % (IQR) | 0.46 (0.33-0.59) | 0.36 (0.25-0.50) | 0.05 |
| Immature reticulocyte fraction, ratio (IQR) | 0.33 (0.29-0.38) | 0.29 (0.25-0.33) | $5\times{10}^{-9}$ |
| Lymphocyte count, 10^9 cells/litre (IQR) | 2.00 (1.61-2.44) | 1.88 (1.50-2.29) | 0.50 |
| Lymphocyte percentage, % (IQR) | 28.86 (23.05-35.03) | 28.45 (23.80-33.30) | 0.48 |
| Mean cell haemoglobin, picograms (IQR) | 30.23 (29.04-31.21) | 31.51 (30.52-32.52) | $5\times{10}^{-11}$ |
| Mean cell haemoglobin concentration, grams/decilitre (IQR) | 34.59 (33.87-35.23) | 34.48 (33.90-35.10) | 0.96 |
| Mean cell volume, femtolitres (IQR) | 87.38 (83.61-89.91) | 91.30 (88.63-93.91) | $4\times{10}^{-15}$ |
| Mean platelet (thrombocyte) volume, femtolitres (IQR) | 9.46 (9.01-10.03) | 9.20 (8.58-9.93) | 0.04 |
| Mean reticulocyte volume, femtolitres (IQR) | 105.28 (102.66-112.24) | 105.80 (101.40-110.48) | 0.20 |
| Mean sphered cell volume, femtolitres (IQR) | 81.84 (78.72-86.17) | 82.64 (79.35-86.09) | 0.62 |
| Monocyte count, 10^9 cells/litre (IQR) | 0.43 (0.31-0.58) | 0.45 (0.37-0.57) | 0.07 |
| Monocyte percentage, % (IQR) | 6.50 (5.21-7.51) | 6.86 (5.60-8.26) | 0.56 |
| Neutrophill count, 10^9 cells/litre (IQR) | 4.00 (3.07-5.47) | 4.04 (3.30-4.99) | 0.29 |
| Neutrophill percentage, % (IQR) | 58.90 (54.38-68.83) | 61.30 (55.80-66.60) | 0.48 |
| Platelet count, 10^9 cells/litre (IQR) | 236.00 (210.88-266.70) | 248.30 (213.90-287.40) | 0.07 |
| Platelet crit, % (IQR) | 0.227 (0.199-0.252) | 0.229 (0.201-0.261) | 0.49 |
| Platelet distribution width, % (IQR) | 16.38 (16.10-16.80) | 16.42 (16.12-16.80) | 0.62 |
| Red blood cell (erythrocyte) count, 10^12 cells/litre (IQR) | 4.77 (4.51-5.03) | 4.50 (4.23-4.79) | $1\times{10}^{-6}$ |
| Red blood cell (erythrocyte) distribution width, % (IQR) | 13.86 (13.29-14.31) | 13.33 (12.90-13.84) | $1\times{10}^{-4}$ |
| Reticulocyte count, 10^12 cells/litre (IQR) | 0.065 (0.051-0.077) | 0.057 (0.043-0.074) | 0.23 |
| Reticulocyte percentage, % (IQR) | 1.377 (1.087-1.619) | 1.255 (0.961-1.619) | 0.60 |
| White blood cell (leukocyte) count, 10^9 cells/litre (IQR) | 6.73 (5.68-8.26) | 6.68 (5.67-7.88) | 0.21 |
| Alanine aminotransferase, u/l (IQR) | 19.62 (15.62-26.17) | 20.16 (15.43-27.43) | 0.58 |
| Alkaline phosphatase, u/l (IQR) | 78.50 (62.80-91.80) | 80.40 (67.30-95.90) | 0.30 |
| Total bilirubin, umol/l (IQR) | 8.79 (7.33-11.31) | 8.08 (6.43-10.42) | 0.06 |

***Supplementary Table 6:*** *Baseline characteristics of 453,688 non-black individuals from the UK general population, presented separately for individuals with HbS/C variants and for non-carriers. Individuals with HbS/C variants were defined as individuals with any combination of HbS and/or HbC variants (HbAS, HbSS, HbAC, HbCC, or HbSC), while non-carriers were defined as individuals without HbS or HbC variants. P-values were calculated using logistic regression for categorical variables and linear regression for continuous variables. no. (%) is displayed for categorical variables while median (interquartile range) is displayed for continuous variables.
no.: number. IQR: interquartile range.*

## Model results for ML models developed and validated on non-Black UK general population individuals

|  | Models developed and validated specifically on non-Black UK general population individuals | | | | | | | | | | | |
| --- | --- | --- | --- | --- | --- | --- | --- | --- | --- | --- | --- | --- |
|  | **Logistic Regression** | | | | **XGBoost** | | | | **Random Forest** | | | |
| ROC-AUC | 0.768 (±0.070) | | | | 0.746 (±0.059) | | | | 0.739 (±0.060) | | | |
| Sensitivity | 98 % | 95 % | 92 % | 90 % | 98 % | 95 % | 92 % | 90 % | 98 % | 95 % | 92 % | 90 % |
| Threshold | 22 | 28 | 34 | 36 | 2 | 4 | 6 | 8 | 28 | 28 | 36 | 38 |
| Specificity | 10 % | 20 % | 34 % | 40 % | 16 % | 23 % | 29 % | 33 % | 23 % | 23 % | 40 % | 44 % |
| TP | 65 | 63 | 61 | 60 | 65 | 63 | 61 | 60 | 65 | 63 | 61 | 60 |
| FN | 1 | 3 | 5 | 6 | 1 | 3 | 5 | 6 | 1 | 3 | 5 | 6 |
| TN | 45.362 | 81.652 | 154.231 | 181.448 | 72.579 | 104.333 | 131.550 | 149.695 | 104.333 | 104.333 | 181.448 | 199.593 |
| FP | 408.260 | 371.970 | 299.391 | 272.174 | 381.043 | 349.289 | 322.072 | 303.927 | 349.289 | 349.289 | 272.174 | 254.029 |

***Supplementary Table 7: Summarized Results for identifying individuals with HbS/C variants Based on Different Fixed Sensitivities (98, 95, 92, 90 %) based on the non-Black UK general population.*** *This table summarizes the performance of Logistic Regression, XGBoost, and Random Forest models at specified sensitivities for non-Black UK individuals. Metrics include the corresponding probability threshold, specificity, and the number of true positives (TP), false negatives (FN), true negatives (TN), and false positives (FP) for each model. The table highlights variations in specificity and risk threshold across models and sensitivity levels. The probability threshold is the probability value that serves as the cut-off for a model's predictions. It determines when the model assigns a specific class label based on its probability estimates. If the probability falls below the threshold, the individual is classified as a non-carrier. Conversely, if it exceeds the threshold, the model predicts that the individual carries HbS/C variants.*

## Study Dataset

***Supplementary Figure 1:*** *Flowchart of the study dataset selection from the UK Biobank*

***Supplementary Figure 2:*** *Flowchart of dataset selection focusing on general population individuals with Black, Black British, Caribbean, African, or other Black ethnic backgrounds from the UK Biobank.*

## Model Development and Validation

***Supplementary Figure 3:*** *Overview of the model development and validation process. The model development and validation process involved creating balanced datasets for training and evaluation. Participants without HbS/C variants (non-carrier individuals) were divided into subsets (blue) of the same size as those with HbS/C variants (red) (see Supplementary Figures 2 and 3). One subset was combined with the HbS/C group to form a balanced dataset (purple) for model development, while the remaining subsets were reserved for validation. This process was repeated for each subset (orange). Three supervised machine learning models (Logistic Regression, Random Forest, and XGBoost) were trained using individuals with HbS/C variants and those without (non-carriers). K-fold cross-validation was applied to enhance model robustness. Performance was evaluated using sensitivity, specificity, the area under the receiver operating characteristic curve (ROC-AUC), and Shapley Additive Explanations (SHAP) to interpret feature importance.*

## Supplementary Results

**Supplementary *Figure 4: Model Performance of XGB Model Developed and Validated on the overall UK general population.****The XGB model's performance metrics for identifying individuals with HbS/C variants are summarized using multiple visualizations. The top-left panel illustrates the sensitivity (blue) and specificity (red) scores across different probability thresholds. The probability threshold is the probability value that serves as the cut-off for a model's predictions. It determines when the model assigns a specific class label based on its probability estimates. If the probability falls below the threshold, the individual is classified as a non-carrier. Conversely, if it exceeds the threshold, the model predicts that the individual carries HbS/C variants. The top-right panel depicts the receiver operating characteristic (ROC) curve, with an area under the curve (AUC) of 0.955 ± 0.006, indicating high model accuracy for identifying individuals with HbS/C variants. The bottom-left bar chart shows the ranked feature importance scores derived from the XGB model, highlighting the most influential variables for prediction. The bottom-right panel presents a violin plot showing the distribution of feature impacts on model predictions, providing additional insight into the model’s interpretability.*

**Supplementary *Figure 5: Model Performance of RF Model Developed and Validated on the overall UK general population.*** *The RF model's performance metrics for identifying individuals with HbS/C variants are summarized using multiple visualizations. The top-left panel illustrates the sensitivity (blue) and specificity (red) scores across different probability thresholds. The probability threshold is the probability value that serves as the cut-off for a model's predictions. It determines when the model assigns a specific class label based on its probability estimates. If the probability falls below the threshold, the individual is classified as a non-carrier. Conversely, if it exceeds the threshold, the model predicts that the individual carries HbS/C variants. The top-right panel depicts the receiver operating characteristic (ROC) curve, with an area under the curve (AUC) of 0.945 ± 0.009, indicating high model accuracy for identifying individuals with HbS/C variants. The bottom-left bar chart shows the ranked feature importance scores derived from the RF model, highlighting the most influential variables for prediction. The bottom-right panel presents a violin plot showing the distribution of feature impacts on model predictions, providing additional insight into the model’s interpretability.*

***Supplementary Figure 6: Model Performance of XGB Model Developed on the overall UK General Population and Validated specifically on the Black UK general population individuals.****The XGB model's performance metrics for identifying black individuals with HbS/C variants are summarized using multiple visualizations. The top-left panel illustrates the sensitivity (blue) and specificity (red) scores across different probability thresholds. The probability threshold is the probability value that serves as the cut-off for a model's predictions. It determines when the model assigns a specific class label based on its probability estimates. If the probability falls below the threshold, the individual is classified as a non-carrier. Conversely, if it exceeds the threshold, the model predicts that the individual carries HbS/C variants. The top-right panel depicts the receiver operating characteristic (ROC) curve, with an area under the curve (AUC) of 0.652 ± 0.018, indicating moderate model accuracy for identifying black individuals with HbS/C variants. The bottom-left bar chart shows the ranked feature importance scores derived from the XGB model, highlighting the most influential variables for prediction. The bottom-right panel presents a violin plot showing the distribution of feature impacts on model predictions, providing additional insight into the model’s interpretability.*

***Supplementary Figure 7: Model Performance of RF Model Developed on the overall UK General Population and Validated specifically on the Black UK general population individuals.****The RF model's performance metrics* *for identifying black individuals with HbS/C variants are summarized using multiple visualizations. The top-left panel illustrates the sensitivity (blue) and specificity (red) scores across different probability thresholds. The probability threshold is the probability value that serves as the cut-off for a model's predictions. It determines when the model assigns a specific class label based on its probability estimates. If the probability falls below the threshold, the individual is classified as a non-carrier. Conversely, if it exceeds the threshold, the model predicts that the individual carries HbS/C variants. The top-right panel depicts the receiver operating characteristic (ROC) curve, with an area under the curve (AUC) of 0.667 ± 0.049, indicating moderate model accuracy for identifying black individuals with HbS/C variants. The bottom-left bar chart shows the ranked feature importance scores derived from the RF model, highlighting the most influential variables for prediction. The bottom-right panel presents a violin plot showing the distribution of feature impacts on model predictions, providing additional insight into the model’s interpretability.*

**Supplementary *Figure 8: Model Performance of XGB Model Developed and Validated specifically on Black UK general population individuals.****The XGB model's performance metrics for identifying black individuals with HbS/C variants are summarized using multiple visualizations. The top-left panel illustrates the sensitivity (blue) and specificity (red) scores across different probability thresholds. The probability threshold is the probability value that serves as the cut-off for a model's predictions. It determines when the model assigns a specific class label based on its probability estimates. If the probability falls below the threshold, the individual is classified as a non-carrier. Conversely, if it exceeds the threshold, the model predicts that the individual carries HbS/C variants. The top-right panel depicts the receiver operating characteristic (ROC) curve, with an area under the curve (AUC) of 0.716 ± 0.017, indicating moderate model accuracy for identifying black individuals with HbS/C variants. The bottom-left bar chart shows the ranked feature importance scores derived from the XGB model, highlighting the most influential variables for prediction. The bottom-right panel presents a violin plot showing the distribution of feature impacts on model predictions, providing additional insight into the model’s interpretability.*

***Supplementary Figure 9: Model Performance of RF Model Developed and Validated specifically on Black UK general population individuals.****The RF model's performance metrics for identifying black individuals with HbS/C variants are summarized using multiple visualizations. The top-left panel illustrates the sensitivity (blue) and specificity (red) scores across different probability thresholds.* *The probability threshold is the probability value that serves as the cut-off for a model's predictions. It determines when the model assigns a specific class label based on its probability estimates. If the probability falls below the threshold, the individual is classified as a non-carrier. Conversely, if it exceeds the threshold, the model predicts that the individual carries HbS/C variants. The top-right panel depicts the receiver operating characteristic (ROC) curve, with an area under the curve (AUC) of 0.714 ± 0.018, indicating moderate model accuracy for identifying black individuals with HbS/C variants. The bottom-left bar chart shows the ranked feature importance scores derived from the RF model, highlighting the most influential variables for prediction. The bottom-right panel presents a violin plot showing the distribution of feature impacts on model predictions, providing additional insight into the model’s interpretability.*

***Supplementary Figure 10****:* ***Model Performance of LR Model Developed and Validated specifically on non-Black UK general population individuals.***  *The LR model's performance metrics for identifying individuals with HbS/C variants are summarized using multiple visualizations. The top-left panel illustrates the sensitivity (blue) and specificity (red) scores across different probability thresholds. The probability threshold is the probability value that serves as the cut-off for a model's predictions. It determines when the model assigns a specific class label based on its probability estimates. If the probability falls below the threshold, the individual is classified as a non-carrier. Conversely, if it exceeds the threshold, the model predicts that the individual carries HbS/C variants. The top-right panel depicts the receiver operating characteristic (ROC) curve, with an area under the curve (AUC) of 0.768 ± 0.070, indicating moderate model accuracy for identifying non-black individuals with HbS/C variants. The bottom-left bar chart shows the ranked feature importance scores derived from the LR model, highlighting the most influential variables for prediction. The bottom-right panel presents a violin plot showing the distribution of feature impacts on model predictions, providing additional insight into the model’s interpretability.*

***Supplementary Figure 11****:* ***Model Performance of XGB Model Developed and Validated specifically on non-Black UK general population individuals.*** *The XGB model's performance metrics for identifying individuals with HbS/C variants are summarized using multiple visualizations. The top-left panel illustrates the sensitivity (blue) and specificity (red) scores across different probability thresholds. The probability threshold is the probability value that serves as the cut-off for a model's predictions. It determines when the model assigns a specific class label based on its probability estimates. If the probability falls below the threshold, the individual is classified as a non-carrier. Conversely, if it exceeds the threshold, the model predicts that the individual carries HbS/C variants. The top-right panel depicts the receiver operating characteristic (ROC) curve, with an area under the curve (AUC) of 0.746 ± 0.059, indicating moderate model accuracy for identifying non-black individuals with HbS/C variants. The bottom-left bar chart shows the ranked feature importance scores derived from the XGB model, highlighting the most influential variables for prediction. The bottom-right panel presents a violin plot showing the distribution of feature impacts on model predictions, providing additional insight into the model’s interpretability.*

***Supplementary Figure 12****:* ***Model Performance of RF Model Developed and Validated specifically on non-Black UK general population individuals.****The RF model's performance metrics for identifying individuals with HbS/C variants are summarized using multiple visualizations. The top-left panel illustrates the sensitivity (blue) and specificity (red) scores across different probability thresholds. The probability threshold is the probability value that serves as the cut-off for a model's predictions. It determines when the model assigns a specific class label based on its probability estimates. If the probability falls below the threshold, the individual is classified as a non-carrier. Conversely, if it exceeds the threshold, the model predicts that the individual carries HbS/C variants. The top-right panel depicts the receiver operating characteristic (ROC) curve, with an area under the curve (AUC) of 0.739 ± 0.060, indicating moderate model accuracy for identifying non-black individuals with HbS/C variants. The bottom-left bar chart shows the ranked feature importance scores derived from the RF model, highlighting the most influential variables for prediction. The bottom-right panel presents a violin plot showing the distribution of feature impacts on model predictions, providing additional insight into the model’s interpretability.*
